# Enterocutaneous Fistula in a Patient with Crohn’s Disease After Internalization of a Foreign Body into the Gastrointestinal Tract

**DOI:** 10.3390/jcm14072327

**Published:** 2025-03-28

**Authors:** Wiktoria Hanna Buzun, Karolina Izabela Pełka, Aleksandra Złotowska, Justyna Łuczak, Dariusz Patkowski, Tomasz Pytrus, Anna Kofla-Dłubacz

**Affiliations:** 1 Student Scientific Group of Gastroenterology, Wroclaw Medical University, 50-368 Wroclaw, Poland; wiktoria.buzun@student.umw.edu.pl (W.H.B.); aleksandra.zlotowska@student.umw.edu.pl (A.Z.); 2 Department of Pediatric Surgery and Urology, Wroclaw Medical University, 50-368 Wroclaw, Poland; justyna.luczak@umw.edu.pl (J.Ł.); dariusz.patkowski@umw.edu.pl (D.P.); 3 2nd Department of Pediatrics, Gastroenterology and Nutrition, Wroclaw Medical University, 50-368 Wroclaw, Poland; tomasz.pytrus@umw.edu.pl (T.P.); anna.kofla-dlubacz@umw.edu.pl (A.K.-D.)

**Keywords:** Crohn’s disease, enterocutaneous fistula, biological treatment, internalization of a foreign body, fistula after ingestion of a foreign body

## Abstract

**Background/Objectives:** Crohn’s disease is a chronic inflammatory condition with periods of exacerbation and remission that can involve any part of the gastrointestinal tract. The basic intestinal manifestation is frequently accompanied by extraintestinal involvement and may lead to complications such as perforations, fistulas and abscesses. Despite Crohn’s disease being the most common reason of intestinal perforation, the other causes should be considered as well. Internalization of a foreign body, although rare, may still occur, especially in the pediatric population. **Methods:** The following case report presents the medical history of an 11-year-old patient who developed an enterocutaneous fistula two years after the diagnosis of Crohn’s disease. Data analysis was carried out on the basis of patient medical records. **Results:** The fistula formed in the course of biological treatment during a period free of other symptoms indicating disease exacerbation. The imaging tests revealed the presence of a foreign body in the gastrointestinal tract, which could have been a potential cause of the observed inflammation that resulted in the development of the fistula. **Conclusions:** The presented case report as well as the literature indicate a correlation between the formation of intestinal fistulas and an active disease process. However, in the absence of symptoms of Crohn’s disease exacerbation, other causes should be considered.

## 1. Introduction

Crohn’s disease (CD) along with ulcerative colitis (UC) belong to the group of inflammatory bowel diseases of autoimmune origin [1]. The condition was first mentioned in 1769 by Giovanni Battista Morgagni [2]. It is a chronic disease characterized by intermittent inflammatory lesions that can localize in any level of the gastrointestinal tract, with the most common location in the ileum terminal [3]. An increasing prevalence has been observed regardless of age group; however, the fastest progression of the disease occurs among pediatric patients [4].

Patients with Crohn’s disease usually present symptoms of malnutrition and chronic abdominal pain accompanied by diarrhea. Other manifestations are related to general inflammation [5]. In the course of the disease, periods of exacerbation and remission usually alternate [6].

The pathogenesis of the disease results from a complex interaction of environmental and immunological factors in patients with genetic susceptibility, leading to damage of the intestinal mucosa [7,8]. The activated cascade of proinflammatory cytokines sustains chronic inflammation and tissue remodeling [9].

Persistent inflammation and damage to the walls of the gastrointestinal tract can lead to local complications such as fistulas [10].

A fistula is defined as an atypical connection formed between a hollow or tubular organ and the body surface or between two hollow or tubular organs, e.g., intestines and body cavities [11]. The development of a fistula is characterized by varied pathogenesis including tissue damage in the course of gastrointestinal diseases as well as traumas [12].

Among the different types of fistulas, internal and external fistulas can be distinguished [13]. Intestinal fistulas are a relatively common complication of Crohn’s disease, as the trans-wall chronic nature of intestinal inflammation causes tissue erosion in adjacent structures [14]. An enterocutaneous fistula (ECF) is an abnormal connection between the intra-abdominal digestive tract and the skin or wound [15]. It forms when existing or newly formed fistulas in the intestines open on the surface of the skin, leading to the secretion of intestinal contents outside the body [16]. ECF’s are associated with a high mortality rate of 6 to 33% due to a predisposition to complications, such as sepsis, malnutrition and substantial skin damage [17]. Treatment of an enterocutaneous fistula depends on its location, severity and the patient’s overall health [18].

It is also possible to develop fistulas caused by ingestion of a foreign body, although they are relatively rare. Internalization of a foreign body into the gastrointestinal tract can happen accidentally, especially among young children [19]. In most cases, foreign bodies pass through the digestive tract without disrupting its normal function and physiology. However, in some situations, especially when it is sharp or large, it can cause damage to the walls of the digestive tract, leading to fistula formation [20].

Untreated fistulas can lead to serious complications, such as infection, ulceration or loss of organ function [21]. Depending on the cause, location and severity, treatment can be based on pharmacological therapy as well as surgical intervention [22].

### 1.1. Case Presentation

An 11-year-old patient with the stricturing and penetrating form of Crohn’s disease was admitted to the clinic in August 2023 for a scheduled dose of adalimumab.

The child had a history of recurrent perianal abscesses treated surgically between the ages of 7 and 9 years. The diagnosis of the disease was established at the age of 9 years in October 2021. According to the standard of management based on the Porto guidelines, a full range of diagnostic tests was performed, including laboratory tests, upper and lower gastrointestinal endoscopy and magnetic resonance enterography [23]. The disease phenotype was determined using the Paris scale of process progression, and the A1a/L1L4/B2B3/p form was diagnosed [24]. At the time of the diagnosis, the patient had a 17 mm-long perianal fistula confirmed by MR examination.

Due to the severe manifestation of the disease, once the diagnosis was established, the patient was qualified for biological treatment. Induction of remission of the disease using chimeric anti-TNF alpha monoclonal antibody (Infliximab) was initiated. Despite the therapy, healing of the fistula was not achieved. Follow-up studies showed the presence of antibodies to the drug in high titers and subtherapeutic levels of the drug. In August 2022, therapy was modified, and a humanized anti-TNF alpha antibody (adalimumab) was included. During that course of treatment, clinical remission of the disease was achieved, and the perianal fistula was healed.

### 1.2. Investigations

After a year of treatment with adalimumab (ADA), during a routine hospitalization for another dose of the drug, massive inflammation with edema of the left inguinal and suprapubic region was found. Laboratory tests showed a moderate increase in C reactive protein (CRP) to 29.5 mg/L and an acceleration of erythrocyte sedimentation rate (ESR) to 44 mm/h. The calprotectin level remained normal and amounted to 63 µg/L. No other abnormalities were found in additional tests.

The ultrasound examination of the cavity showed swollen subcutaneous tissue with increased and blurred echogenicity, with fluid collection in the examined area and lymphadenopathy. The image of the wall of the large intestine and the terminal ileum was without signs of inflammation.

**Figure 1:**
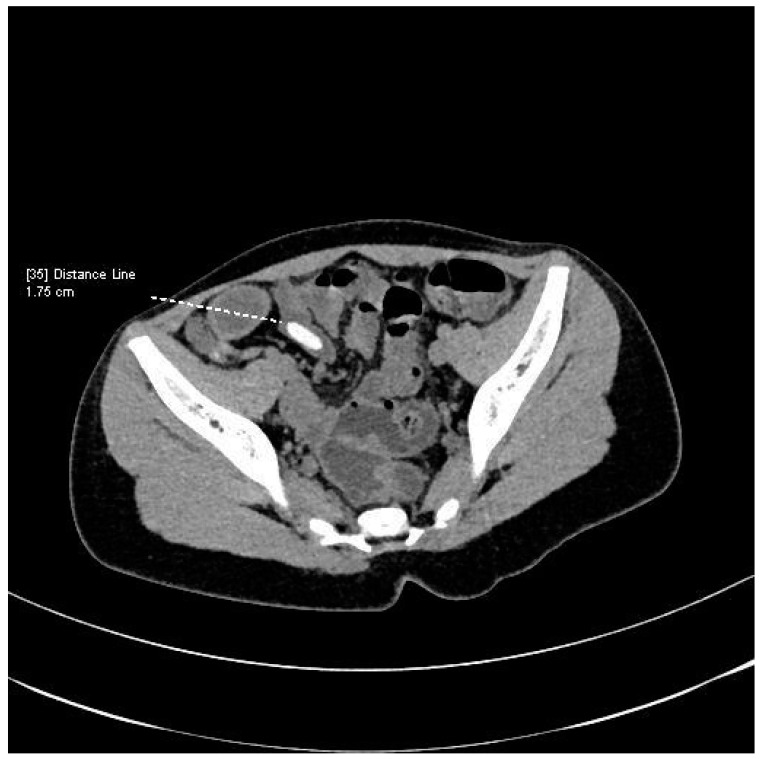
CT scan of the abdomen and pelvis.

**Figure 2:**
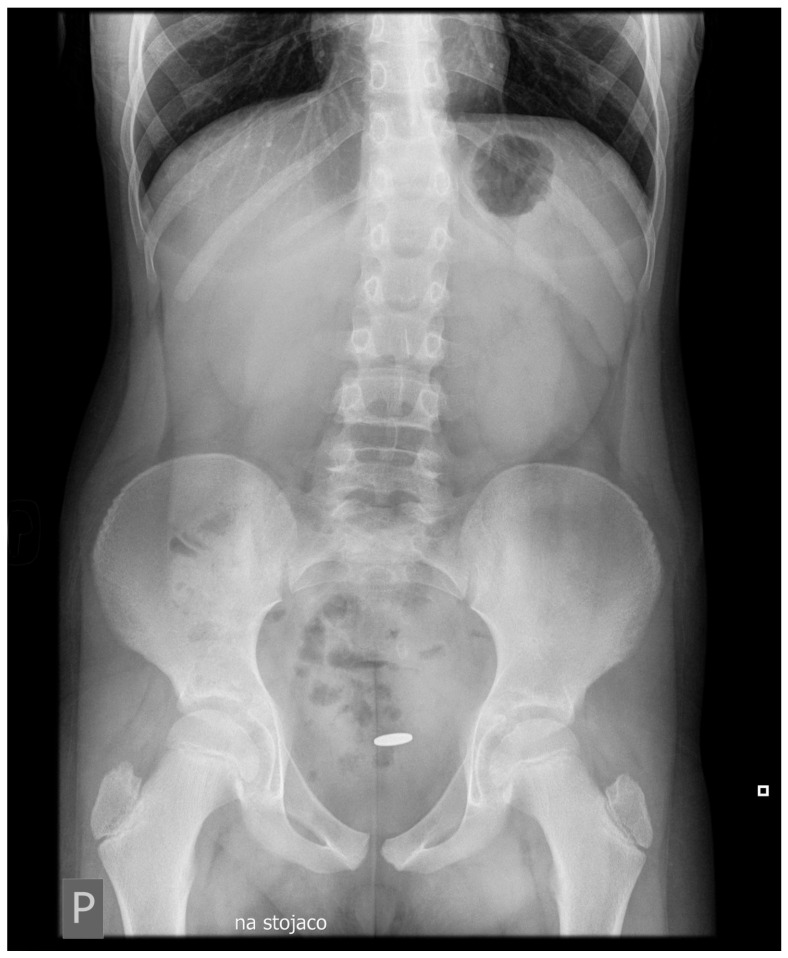
Abdominal X-ray with visualization of the foreign body.

A CT scan of the left suprapubic and inguinal region showed extensive swelling of the subcutaneous tissue (47 × 37 mm in the transverse section, 60 mm in the sagittal section). Additionally, dorsally from the left abdominal oblique muscle and anteriorly from the bladder, a tubular structure crossing the seminal cord suggestive of an active intestinal fistula was observed [Figure 1]. The examination also showed a metallic coin-like structure measuring 18 × 7 mm in the sigmoid projection of the evacuated loop of the small intestine [Figure 2]. At the time, the patient admitted that he had swallowed a coin a year ago. The patient underwent endoscopic examination without visualizing a foreign body in the large intestine, and the mucosa on the viewed section was without features of inflammatory disease.

The patient was surgically consulted, a date for elective surgery was set, and pharmacological treatment was continued under control of inflammatory parameters and clinical status. On the 9th day of treatment, evacuation of the abscess content into the surrounding tissues occurred. An emergency surgery was performed immediately.

During the procedure, the intestines appeared free of inflammation. The entire length of the small intestine was inspected from the cecum to the ligament of Treitz. A 20 cm segment from the cecum revealed a protrusion in the intestinal wall, suggesting the presence of a coin. No abnormalities were found in the descending colon, sigmoid colon or rectum. The bowel was incised, and the coin was removed. The left groin wound was cleaned of necrotic tissue.

There was a further course of treatment without complications. After healing of the postoperative wound, the patient was admitted again to the Department of Gastroenterology for clinical re-evaluation. In laboratory tests, inflammatory markers were negative, and blood account parameters were normal; moreover, the abdominal ultrasound image was normalized. During subsequent follow-ups, the patient remained in clinical, laboratory and endoscopic remission. The biological treatment was discontinued. An evaluation 24 months after the surgical intervention showed no progression of the disease. The changes in inflammatory markers during the course of treatment are summarized in Table 1.

## 2. Discussion

A fistula may occasionally be described as the first manifestation of Crohn’s disease [25].

A systematic review by Liesbeth Jozefien Munster et al. found that perianal fistulas occur as the first manifestation of CD in 8.6% of all patients with CD. However, the percentage of patients with a perianal fistula (PAF) as the first manifestation was higher among adult patients compared to pediatric patients. The time required to establish the diagnosis of CD among patients with a PAF as the first symptom is significantly longer than among patients reporting gastrointestinal lumen complaints [26].

Similarly, a prospective analysis of data on the course of disease of patients operated on from January 2008 to January 2017 for the presence of an anal fistula at Picardi University in Amiens, France, showed that 7% of the operated group was diagnosed with CD [27].

In the General Surgery Department of the University of Minnesota, USA, the case of a 59-year-old woman who developed a tubo-ovarian abscess that led to the formation of a non-healing ECF was described. The patient was diagnosed with Crohn’s disease 2 years later [17].

A study carried out at the Medical University of Lublin, Poland, describes five cases of patients aged 13–16 years referred to the Department of Pediatric Gastroenterology in 2017–2019, who were diagnosed with anorectal fistulas. Three patients presented perianal abscesses drained by fistulas. All patients were eventually diagnosed with Crohn’s disease.

Similarly, in the patient who is the subject of this case report, a perianal abscess-like lesion that finally led to the development of a fistula had already been managed before the disease was diagnosed. Lesions of this type usually correlate with an active disease process. As a result of the biological treatment, the patient was in a remission period of the CD, which was indicated by clinical symptoms and laboratory results [5,14,28].

There have been numerous meta-analyses focusing on evaluating the efficacy of biological treatment in CD. B. Barberio et al. demonstrated the superiority of infliximab in inducing clinical remission over other biologic drugs. In case of a loss of response to infliximab, the introduction of adalimumab is recommended [29,30]. Loss of efficacy of biologic treatment is often associated with the production of antibodies against the biologic agents. These lead to a decrease in the serum concentration of the drug and block its proper action, resulting in the inability to achieve and maintain remission [31].

S Gómez-Senent et al., after retrospectively analyzing 26 patients with CD who presented an ECF fistula in three tertiary centers in Spain, stated that anti-TNF alpha treatment enhanced ECF drainage in 64%, making it a potential pre-surgery treatment strategy in a specific group of patients [32].

However, cases of unsuccessful closure of perianal fistulas despite therapy have been described during ADA biological treatment. A cross-sectional study of patients with CD-related perianal fistulas treated with ADA was conducted in four French hospitals between December 2013 and March 2018. The study cohort included 34 patients, of whom only fifteen patients achieved clinical remission (44%) and only four patients had signs of fistula healing on MRI [33].

Similarly, in the case of enterocutaneous fistulas in CD, in the study by K. Fujiwara et al., the introduction of ADA treatment in three patients with a current enterocutaneous fistula in the course of the disease led to its closure in only one patient [34].

However, despite the fact that fistulas are a typical and relatively common symptom of Crohn’s disease, it seems reasonable, each time they occur, to consider the involvement of additional factors in the etiopathogenesis. Indeed, the lack of a thorough differential diagnosis can lead to diagnostic confusion and unnecessary modification of therapy from which the patient will not benefit. The Yale Journal of Biology and Medicine describes a case of Crohn’s disease involving the large intestine with foreign body ingestion resulting in the formation of a cologastric fistula. The presence of the ingested foreign body likely resulted in the development of a fistula, which was rare for CD and resistant to conservative treatment [35].

In some cases, the initial diagnosis of Crohn’s disease was incorrectly made in patients in whom the presence of an enterocutaneous fistula was identified with the first symptom of the disease, while internalization of a foreign body in the gastrointestinal tract led to its formation [36,37,38].

The literature describes only a few cases of Crohn’s patients in whom a foreign body caused a fistula. It is more common to encounter cases of misdiagnosed Crohn’s disease in patients in whom a foreign body in the gastrointestinal tract mimicked the disease. A summary of other examples described in the literature is provided in Table 2.

Moreover, in this type of case, the analysis of biochemical parameters, including inflammatory markers such as CRP, procalcitonin or white blood cell, has limited value in differentiating the etiology of perforations, abscesses or fistulas. However, they play a significant role in assessing the dynamics of the tissue process, regardless of its initial cause, and are the basis for therapeutic decisions, including surgical intervention [39].

The measurement of fecal calprotectin, a marker with significant sensitivity in detecting the inflammation in the distal gastrointestinal tract, also has limitations, especially in relation to Crohn’s disease with proximal localization of the lesions [40]. In our patient, the fecal calprotectin level was not elevated and reached 63 µg/g. There was no significant increase in other inflammatory parameters as well. In such cases, the determining examinations are an endoscopic assessment of the gastrointestinal tract and imaging tests.

Depending on the type of foreign body, the degree of tissue damage may vary. Most foreign bodies pass freely through the gastrointestinal tract, approximately 20% of cases require endoscopic removal, and only approximately 1% of patients require surgical intervention. The guidelines for the removal of foreign bodies from the gastrointestinal tract has been published, among others, by the European Society for Pediatric Gastroenterology Hepatology and Nutrition (ESPGHAN) [41]. Endoscopic procedures are the preferred method for foreign body removal. Furthermore, the new endoscopic technique of device-assisted enteroscopy may be considered in the diagnostic process when available [42]. However, due to the potential for Crohn’s disease strictures and limited availability of small-bowel enteroscopy in pediatric patients, surgical intervention is often required [43].

Urgent intervention is necessary in the case of batteries, magnets and sharp objects causing chemical, thermal or mechanical damage with subsequent tissue necrosis [44]. Neutral, round bodies of small size should pass freely through the digestive tract, but in the case of a coexisting disease changing the conditions in the lumen of the digestive tract, this process can be disrupted, which predisposes the individual to the development of a perforation and the possible formation of a tissue abscess or fistula [45]. It seems that the prolonged retention of the foreign body in the presented patient may have been related to the occurrence of the described complication, although the impact of the inflammation by itself cannot be eliminated [46]. The further follow-up and long-term remission of Crohn’s disease after surgery seem to confirm that mechanism.

**Table 2:** Summary of case reports involving internalized foreign bodies in patients with suspected/diagnosed Crohn’s disease.

foreign objects causing fistulisations in Crohn’s patients	Crohn’s disease with impaction of the 20-pence coin in a distal terminal ileum [47].
foreign objects mimicking Crohn’s disease	Toothpick ingestion causing an ileum fistula [48]; An ileo-cecal foreign body granuloma [49]; Synthetic plastic packaging causing an ileo-cecal junction perforation [50]; Pen ingestion mimicking Crohn’s symptoms [51]; Plastic straw disguising as Crohn’s [52]; Chicken bone ingestion mimicking inflammatory bowel disease [53]; Ileal perforation from foreign body granuloma [54].

The presented case of a patient who developed an enterocutaneous fistula in the course of Crohn’s disease during endoscopic and laboratory remission achieved by biologic treatment and with internalization of a foreign body into the gastrointestinal tract is the starting point of the discussion. What was the cause of the patient’s condition? Is it due to the ongoing disease process or is it a consequence of internalization of the foreign body?

In the patient’s two-year follow-up after the surgical treatment, there were no signals suggesting CD recurrence. Despite the decision not to continue biological therapy, the patient remained asymptomatic, which supports the predominant involvement of the foreign body in the process of fistula formation.

## 3. Conclusions

The presented case demonstrates the need to consider causes other than Crohn’s disease in the differential diagnosis, which, however, remains one of the most common causes of fistula-like lesions. Misclassification can result in unnecessary intensification of treatment in a situation with a controlled inflammatory process.

## Figures and Tables

**Table 1:** Summary of inflammatory marker changes during hospitalization.

	Diagnosis	On Admission	During Hospitalization		Operation Day	Follow-Up	
	10.2021	09.2023	09.2023	10.2023	10.2023	11.2024	03.2025
Calprotectin [µg/g]	796	-	63	-	-	92	132
CRP [mg/L]	17	29.5	10	2.5	6.5	<0.5	<0.5
WBC [×10^3^/µL]	10.53	9.43	7.01	6.92	-	6.51	5.05
ESR [mm/h]	39	44	-	-	-	7	8
Procalcitonin [ng/mL]	-	-	-	-	0.02	-	-

## Data Availability

Data are contained within the article.

## Abbreviations

The following abbreviations are used in this manuscript:

MDPI	Multidisciplinary Digital Publishing Institute
ADA	Adalimumab
ECF	Enterocutaneous fistula
PAF	Perianal fistula
CD	Crohn’s disease
US	Ulcerative colitis
CT	Computer tomography
MR	Magnetic resonance
